# GenoLink: a graph-based querying and browsing system for investigating the function of genes and proteins

**DOI:** 10.1186/1471-2105-7-21

**Published:** 2006-01-17

**Authors:** Patrick Durand, Laurent Labarre, Alain Meil, Jean-Louis Divo1, Yves Vandenbrouck, Alain Viari, Jérôme Wojcik

**Affiliations:** 1 Hybrigenics SA, 3–5 Impasse Reille, 75014 Paris, France; 2 AGC, UMR CNRS 8030 – Genoscope, 2 rue Gaston Crémieux, 91000 Evry, France; 3 Genome Express, 11 Chemin des Prés, 38944 Meylan, France; 4 INRIA Rhône-Alpes, 655 Avenue de l'Europe, 38334 Saint-Ismier Cedex, France; 5 IRISA-INRIA, Campus de Beaulieu, 35402 Rennes Cedex, France; 6 DRDC/BIM, CEA-Grenoble, 17 Avenue des martyrs, 38054 Grenoble Cedex 9, France; 7 Serono Genetics Institute, Route Nationale 7, 91030 Evry Cedex, France

## Abstract

**Background:**

A large variety of biological data can be represented by graphs. These graphs can be constructed from heterogeneous data coming from genomic and post-genomic technologies, but there is still need for tools aiming at exploring and analysing such graphs. This paper describes GenoLink, a software platform for the graphical querying and exploration of graphs.

**Results:**

GenoLink provides a generic framework for representing and querying data graphs. This framework provides a graph data structure, a graph query engine, allowing to retrieve sub-graphs from the entire data graph, and several graphical interfaces to express such queries and to further explore their results. A query consists in a graph pattern with constraints attached to the vertices and edges. A query result is the set of all sub-graphs of the entire data graph that are isomorphic to the pattern and satisfy the constraints. The graph data structure does not rely upon any particular data model but can dynamically accommodate for any user-supplied data model. However, for genomic and post-genomic applications, we provide a default data model and several parsers for the most popular data sources. GenoLink does not require any programming skill since all operations on graphs and the analysis of the results can be carried out graphically through several dedicated graphical interfaces.

**Conclusion:**

GenoLink is a generic and interactive tool allowing biologists to graphically explore various sources of information. GenoLink is distributed either as a standalone application or as a component of the Genostar/Iogma platform. Both distributions are free for academic research and teaching purposes and can be requested at academy@genostar.com. A commercial licence form can be obtained for profit company at info@genostar.com. See also .

## Background

The development of genomic and post-genomic technologies is producing a large amount of heterogeneous data for investigating the functions of the genes and their products. Biological data are spreaded across an increasing number of databases that differ widely in terms of quality, consistency, diversity and availability [1]. The biologists are now faced with the problem of analysing this information and turning it into new knowledge. Simple analysis is usually performed directly on a database through a query language (usually SQL for relational databases) or using a pre-defined set of queries. More sophisticated analyses require to integrate heterogeneous data coming from various sources and rely upon the use of specialized algorithms and data structures, since database models are not suitable for direct algorithmic use.

**Figure 1:**
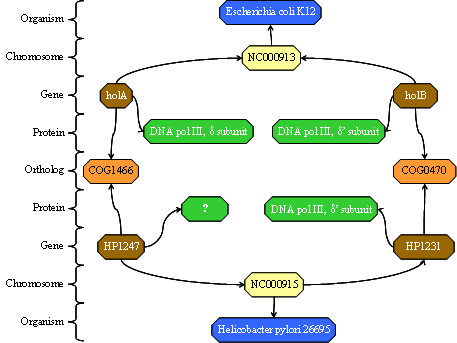
***Example of a graph representing biological data and how it can be used to infer new information***. The graph vertices are represented by boxes and are associated to biological entities (Organism, Chromosome, Gene etc). The edges (arrows) represent the relationships between these entities (Chromosome *BelongsTo *Organism, Gene *IstranslatedTo *Protein). On this example, genes *holA *and *HP1247 *are known to be orthologs (COG1466) but the protein product of HP1247 is not annotated in the sequence databank (RefSeq). The graph suggests to annotate the product of *HP1247 *as the delta subunit of polymerase III.

An efficient way to analyse the functions of the genes/proteins consists in exploring the relationships between various kinds of biological data [2]. As a very simple example, it is possible to assign a function to a protein from a given organism knowing that this protein is encoded by a gene which is ortholog to another gene encoding a well-known protein in another organism (Figure 1). In that case, a network of objects (genes, proteins and organisms) is actually explored using relationships (a gene 'belongs' to an organism, a gene 'encodes' a protein and a protein 'is similar' to another one).

More generally, such a network can be modelled as a graph. A graph is defined by two sets (V,E) where V is a set of vertices and E is a set of edges. A vertex represents an object in the network (see Figure 1). An edge connects two vertices and corresponds to a particular relationship in the network (see Figure 1). A graph is said to be directed (resp. undirected) if it is exclusively made of oriented (resp. not oriented) edges. Graphs benefit from efficient algorithms and are widely used in computer science. During the past few years, graph theory has been used in biology for data modelling purpose, especially for molecular interaction databases (e.g. IntAct, [3]) and metabolic pathway databases (see [4] for a review). Graph-based algorithms have also been used to answer various biological questions, such as in the field of protein-protein interactions networks (e.g. [5]) or biochemical networks (see [6] for a recent review). Most of the current solutions are dedicated to restricted set of data and/or particular analyses, and they cannot be easily modified to accommodate new data/methods. Another approach to target data graph analysis in a more general way consists in using a graph database. Significant systems like GOOD [7], Hy+ [8], Gql [9], Hyperlog [10] and the system from Butler et al. [11] provide visual interfaces and pattern-oriented query languages allowing the end-user to answer various biological questions through the use of diagram-based queries. This approach is more intuitive and more generic than the previous ones, it can be applied to various data types, and does not require the design of particular algorithms to answer particular questions. However, current graph database systems have a limited data modelling power since they rely on a flat (i.e. non hierarchical) data model. Graph querying approach could greatly benefits from object-oriented data modelling techniques since they provide a higher level of abstraction (especially through objects inheritance) that is especially well-suited to represent complex biological data. In this context, the Snow system (under development, see [12]) provides an environment dedicated to the representation and analysis of biological networks which is based on an entity-relationship data model.

This work is concerned with the development of GenoLink, a generic software application dedicated to the exploration of graphs, where vertices and edges are enriched with data modelled using an entity-relationship model. GenoLink can be seen either as a generic graph querying and browsing engine or as a dedicated application for biologists. From the first point of view, GenoLink provides a generic graph data structure, a graph query engine, allowing to retrieve sub-graphs from the entire data graph, and several graphical interfaces to express such queries and to further explore their results. It is important to note that the graph data structure does not rely upon any particular data model but can dynamically accommodate for any user-supplied data model. However, since our primary goal concerns genomic and post-genomic applications, GenoLink is distributed with a default data model for this particular purpose.

## System and methods

### Overview

**Figure 2:**
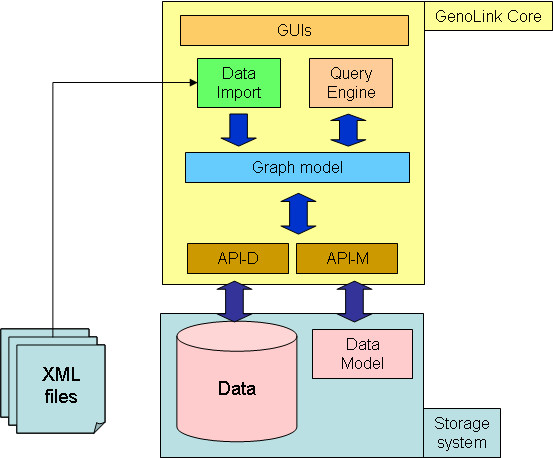
***GenoLink overall architecture***. The GenoLink core provides the generic graph data structure on the top of which querying and display operations are performed. It is separated from the storage system and interacts with it through an API based on an entity-relationship model. Data can be imported in two ways into the system: 1) through the storage system or through external XML files. (See text for more details).

The overall architecture of GenoLink, represented on Figure 2 is composed of two main components : the core and the data storage system. The core provides the graph representation of the data on the top of which querying and display operations are performed. It interacts with the storage system through an API based on an entity-relationship model (like UML). This API basically allows data retrieval, creation or modification. The core relies on a simple and generic graph model made of two entities: vertices and edges. In order to link them to actual data, vertices and edges have two attributes: a data identifier (ID) and a data type. The ID is a unique identifier to a piece of data kept in the storage system (e.g. a gene), and the data type identifies the corresponding type for this data. The API is therefore composed of two parts (Figure 2): one to access the data (API-D), and one to access the data model (API-M).

Accessing the data model has several important consequences to the functionalities of the core. First, the data model can specify that data types are organized into hierarchies (i.e. classes and subclasses in an object-oriented model). In that case, the core engine will retrieve this information (through the API-M) and can further use it in the querying process. For instance, if, during a query, the user requires that a vertex should be of type 'Gene' and if 'ProteinGene' is a subtype of 'Gene', then all data of type 'ProteinGene' should match this vertex as well. A second aspect relates to data consistency. By querying the model (through API-M) the core can easily ensure that a user query is consistent with the data model, for instance that types are connected through the proper relationships. Finally, a last aspect relates to the content of the data itself. A piece of data in the storage system is described by an identifier (the ID) and a set of attributes. For instance a gene may have attributes to describe its name, its description and its length. During a query, some constraints will be ascribed to these attributes (for instance "length > 1000"). Again, by querying the data model, the core is able to control the consistency between the user's constraints and the data model.

In this architecture, any kind of data storage system could, in theory, be used, as long as it can accommodate the API-M and API-D. In practice, we implement GenoLink by using an object-oriented DBMS (OO-DBMS), based on the AROM system [13]. The reason for using an OO-DBMS is that the mapping between the generic graph model and the object model is greatly simplified.

### Data connection and data import

There are basically two mechanisms to transfer data sets from external sources into the GenoLink system: data connection and data import.

Data connection is straightforward: when the core is started, it connects trough the API-D to the storage system and constructs a 'shallow graph' representation of this data. We use the term 'shallow graph' to point out that the graph model does only retain in memory the graph topology and the identifiers and data types attached to the vertices and edges, not the whole data set itself. Indeed, when a specific part of data is needed (e.g. the value of some attributes) the core will dynamically request it to the storage system through API-D.

The data connection mode is conceptually simple but has the drawback that all the data should be already available in the storage system. This does not allow a great flexibility for the user to add some specific data. To this purpose, GenoLink provides a data import mechanism. In this mode the data are described in one or more external XML-formatted flat files. Such a file contains the description of a data graph in terms of vertices and edges. Each XML vertex (or edge) should specify a data type and an ID. When loading the file, the core first checks that the provided data type is known (through API-M). Then it checks whether the ID already exists. If it does not, a new piece of data (object) will be created in the storage system (through API-D). The XML vertex (or edge) may also contain attributes. Again, the core checks that each provided attribute is correct for this type and will assign the provided value to it (unless the attribute was already valuated). This mechanism provides a flexible way of instantiating the data into the storage system since several pieces of information pertaining to the same object can be progressively put together by successively loading external files. For instance, genomic information can be first loaded to instantiate genes together with their relationships to an organism, and additional information, such as their homology relationships or their involvement in metabolic processes, can then be added. Of course this integration process relies on using the same identifier to designate the same object in the various files.

In the context of genomic and post-genomic applications, GenoLink is distributed with a set of parsers and XSL transformation sheets to facilitate the construction of these XML files, as described later.

### Data Exploration

Exploring a data graph consists in finding vertices or paths between two vertices or, more generally, sub-graphs of particular interest. With currently available applications, this can be done with dedicated graph algorithms (e.g. [14]) or constraint programming systems (e.g. [15]; for a recent review on graph matching, see [16]). However, GenoLink proposes an exploration mechanism based on a graphical 'query/browse' system adapted to data graphs.

In GenoLink, exploring a data graph is done in two steps. First, the user formulates a query on the data graph. The results of such a query are sub-graphs representing portions of the whole data graph of particular interest. Then, the user graphically browses into the whole graph by using these sub-graphs as starting points.

The creation of a query can be achieved in two ways: either using a dedicated graphical user interface, or using a query language (GenoLink Query Language, or GQL). The former does not require any programming skills.

**Figure 3:**
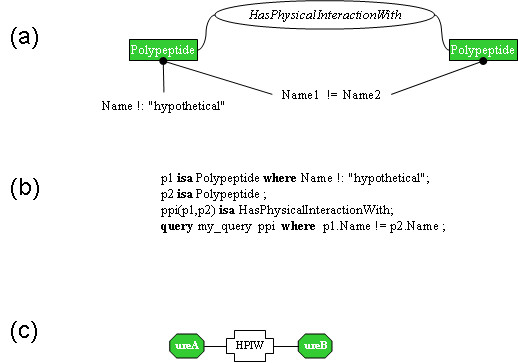
***Example of a GenoLink query***. (a) A simple query represented as a graph pattern. This simple query will retrieve hetero (Name1 != Name2) protein-protein interactions where at least one of the two proteins has an annotated known function (Name !: "hypothetical"). (b) The GQL script describing the same query. GQL reserved keywords are indicated in bold. In the declaration of variable *p1*, the expression located to the right of the 'where' clause is a local constraint (here: the name must not contain *hypothetical*). In the declaration of *my_query*, the expression located to the right of the 'where' clause is the global constraint (here: the two names must be different). (c) Result obtained by executing this query against the data graph shown in Figure 4b. When applied to the entire *Helicobatcer pylori *strain 26695 data set, this query yields 896 different answers.

Formally, a GenoLink query is a graph pattern where vertices and edges are made of the data types defined by the data model (Figure 3). These vertices and edges may carry local constraints consisting of algebraic expressions involving the vertex or edge attributes. A query may also define a global constraint consisting in algebraic expressions involving attributes of different vertices or edges. An occurrence of a graph pattern in the data graph is a sub-graph of this data graph where the vertices and the edges fully satisfy the graph pattern constraints: topology, data type (or subtypes) of vertices and edges and constraints on attributes.

In addition to query declaration, GenoLink is also able to compute union, difference or intersection between sub-graphs. A full description of querying and graph operation capabilities of GenoLink are provided with the documentation distributed with the software.

#### The matching algorithm

The search engine of GenoLink is responsible for searching for all matches of a graph pattern (hereafter called the query graph) against the whole data graph. This graph search problem is related to the sub-graph isomorphism problem, which is known to be NP-complete [17]. One of the most commonly used algorithms to solve that problem is the backtracking algorithm proposed by Ullmann [18]. The algorithm used by GenoLink is inspired from the Ullmann's one but present some slight differences that mostly come from the fact that, in the sub-graph isomorphism problem, one has to compare two graphs of the same kind whereas, in the graph pattern problem, the nature of the vertices and edges is not strictly the same between the graph pattern and the data graph. Nevertheless, due to the close similarities between the two problems we (abusively) state that a result sub-graph is 'isomorphic' to the pattern graph.

GenoLink relies on a depth-first search (DFS) approach which is guaranteed to find all the ways a query graph matches the data graph. Local and global constraints are used in the algorithm in order to prune the search space. More precisely, the algorithm uses a two-steps process. First, it uses the data types declared in the vertices and edges of the query graph to find out which one returns the smallest number of vertex/edge instances from the data graph. That particular vertex or edge will be used as a seed for the DFS exploration. The DFS proceeds through the data graph, using the query graph as a guide, and will progressively add vertices and edges into the nascent sub-graph. At each step, data types and local constraints are checked to prune the search. Finally, each time a sub-graph has been completed, the global constraint is applied.

At the end of the DFS, an additional filtering step may optionally be added in order to discard redundant result graphs (such redundancies may arise from symmetrical relationships). This step is similar to the use of a *distinct *clause in SQL queries.

### Implementation

#### Default DBMS

As mentioned earlier, GenoLink comes with a default storage system implemented using the AROM (Associating Relations and Objects for Modelling, [13]) Java-based system. AROM is an entity-relationship knowledge modelling and management system freely available (see [19]). It provides data management services: formal description of the data model, creation and modification of instances and data persistence. It has a richer metamodel than GenoLink that makes it easy to connect to the API. On the other hand, it has the drawback of keeping all the instances in memory that limits its usage to the available RAM (see the Results section for order of magnitude of the RAM required). In the future we plan to extend GenoLink to other OO-DBMS, to XML storage systems or to Relational DBMS. The later case is the most difficult since the metamodels are quite different and will probably require a simplification and refactoring of the API.

#### Default data model

**Figure 4:**
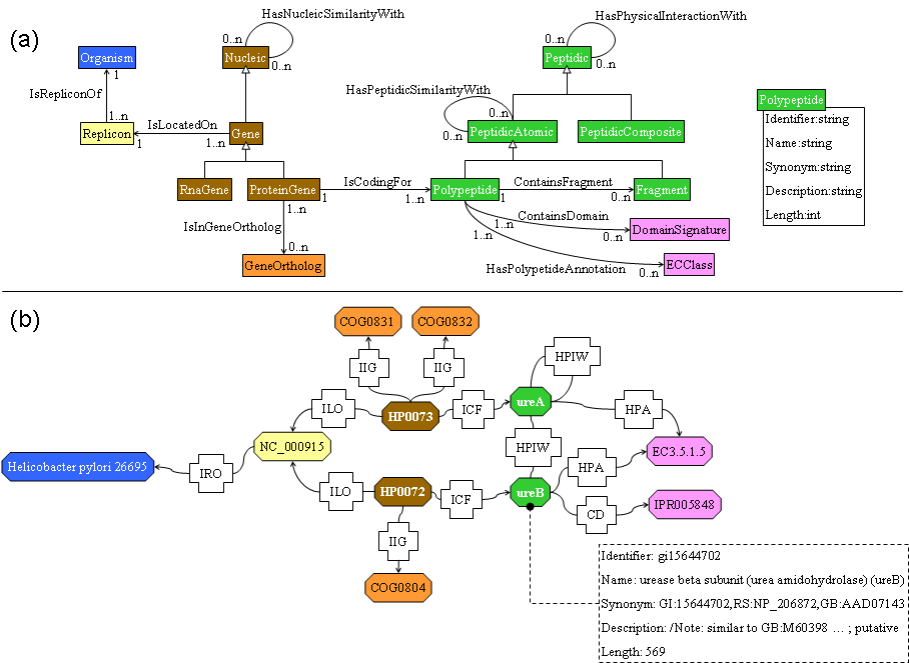
***GenoLink default data model and example of a data graph***. (a) Excerpt of the UML diagram describing the main classes and associations of the default data model provided with GenoLink. Classes are indicated by boxes (white arrows indicate inheritance) and association names are indicated in italics. For clarity, class and association attributes have not been indicated (an example is shown to the right part of the figure, with the Polypeptide class). The complete diagram is distributed with the GenoLink software documentation. (b) An example of data graph based on this data model. It represents a portion of the genome of the bacterium *Helicobacter pylori *strain 26695 (NCBI RefSeq entry no. NC000915); IRO, ILO, ICF, IIG, HPA, CD and HPIW stand for edges that are instances of associations: IsRepliconOf, IsLocatedOn, IsCodingFor, IsInGeneOrtholog, HasPolypeptideAnnotation, ContainsDomain and HasPhysicalInteractionWith. The entire data graph for this genome actually contains 3197 vertices (1 Organism, 1 Replicon, 1576 ProteinGenes, 43 RNAGenes, 1576 Polypeptides) and 4664 edges (1 IsRepliconOf, 1619 IsLocatedOn, 1576 IsCodingFor and 1468 HasPhysicalInteractionWith). The dashed box displays the attributes for the Polypeptide ureB. COG, EC and IPR data are from the COG database [21], the Enzyme Commission database [24], and the InterPro database [23], respectively. Protein-protein interactions are public data available from Hybrigenics [30] and distributed with GenoLink.

GenoLink comes with a default data model (implemented in AROM) targeted at representing microbial genomics and functional genomic data. The data model comprises 25 classes and 28 associations. A UML representation of the main classes and associations is depicted on Figure 4. There are basically three main categories of classes: one for representing nucleic biological entities (e.g. Gene); one for representing proteic biological entities (e.g. Polypeptide), the later being typically linked to the former by the 'isCodingFor' association. The last category relates to sets of entities like functional classifications. A typical example is a gene orthology classification (like COG) or the EC number classification for proteins. Additional specific associations allow representing the results of particular experiments. A typical example, depicted on Figure 4, is the 'HasPhysicalInteractionWith' association between peptidic entities which allows representing the results of protein-protein interaction experiments.

The default data model can be easily modified or, even, completely replaced by another one, in order to accommodate other experiments or different biological problems. This is done by editing the AROM model text file. Thanks to the API-M, the GenoLink core will dynamically adapt to the modified or new data model.

#### Default data import

Together with the default data model, GenoLink provides a set of default data parsers for popular data formats: the genomes from the RefSeq division of GenBank [20], the NCBI COG database of pre-computed clusters of orthologous genes and its functional classification [21], the Gene Ontology (GO) classification [22], the InterPro domains database [23], the Enzyme Commission (EC) classification [24], and protein-protein interactions formatted using HUPO PSI-MI [3].

The purpose of these parsers is to produce (or transform) files coming from the above mentioned data bases into GenoLink XML-formatted files. These files are then loaded on the fly by the XML import module as previously described. Of course these XML files fit to the default data model. If the user changes this model, then the parsers should be modify accordingly.

#### User interfaces

GenoLink comes with various viewers/editors, namely the KB Rider, the Annotator, the Query Builder, the Table Rider and the Graph Rider.

The KB Rider is responsible for displaying the data model. By using the API-M it allows to browse the classes and associations that have been defined in the model. In a similar way, the Annotator allows to browse and edit the data actually attached to vertices and edges.

**Figure 5:**
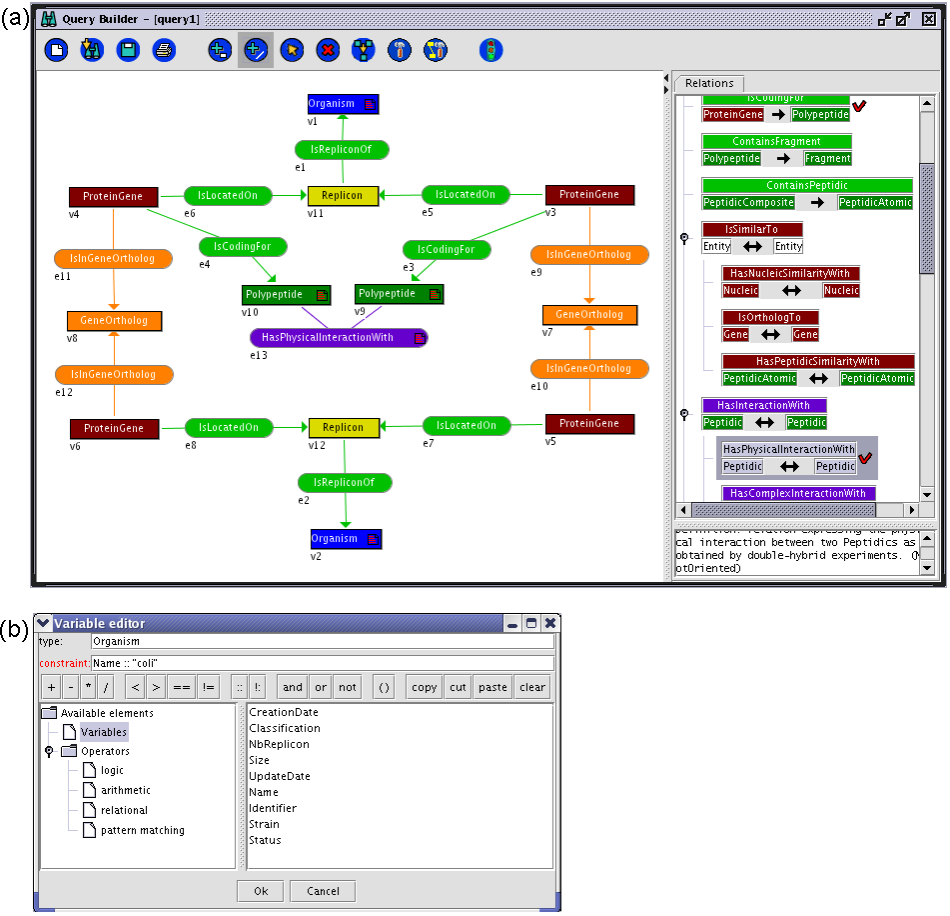
***The Query Builder: a graphical user interface to create a query***. (a) Main window. This snapshot shows the interactive construction of query Q7 presented in Table 1 [see Additional file 1]. The left panel displays the graph pattern being constructed. The right panel displays either the hierarchy of classes or the hierarchy of associations of the data model. Here the user is adding an association therefore the hierarchy of associations is shown. The associations with non empty set of instances are marked "V". (b) Clicking on a vertex or edge will popup this constraint editor to add an algebraic constraint on the corresponding object. Here the name of the organism (represented by vertex v2) should match "coli".

The Query Builder allows to graphically create a query graph (i.e. without knowledge of the GQL language). A typical screenshot is displayed on Figure 5. As shown in Figure 5a, the user can compose the query graph by selecting vertices (and edges) in a window (right part of the figure). Clicking on a vertex (or edge) will call the constraint editor as shown on Figure 5b, allowing to add an algebraic constraint on the corresponding object.

**Figure 6:**
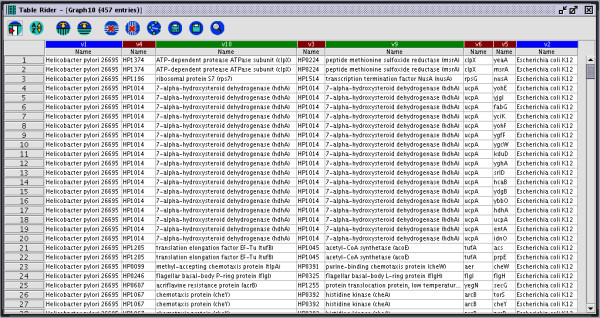
***The Table Rider: a graphical interface to display the query results in a tabular form***. The results of the execution of query in Figure 5 are displayed in a tabular form. Each row of the table corresponds to a result sub-graph. Each column's header contains two lines: the first one indicates the label of a vertex from the query graph (see Figure 5); the second line indicates the name of an attribute of this vertex (the user can select which attributes to display: in this example only the "Name" attributes have been selected).

**Figure 7:**
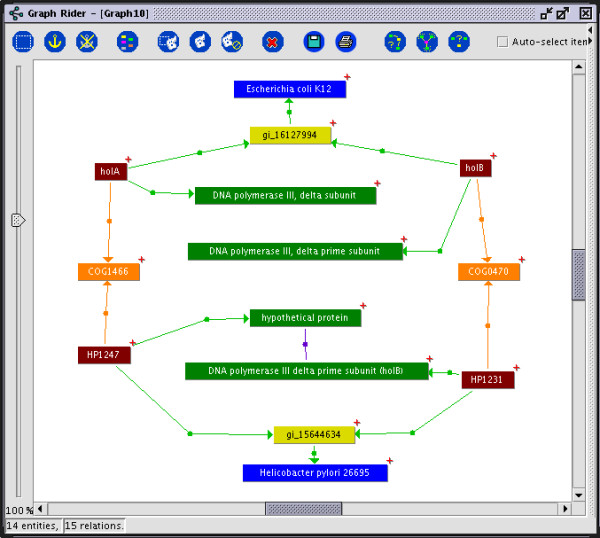
***The Graph Rider: a graphical interface to display the query results in a graph form***. By selecting a line in the Table Rider (Figure 6), the user can display the actual sub-graph associated to it. This snapshot shows an example of result sub-graph corresponding to the Query Q7 (Table 1 [see Additional file 1] and Figure 5). The edge linking the two *H. pylori *Polypeptides corresponds to a physical interaction (HPIW). The red crosshair on the top-right of some vertices denotes that they are linked to some others that are not currently shown. These vertices may therefore be further expanded to gain more information about the full data graph. In this example, this operation has been performed on vertices holA and holB (from *E. coli*) in order to display the corresponding Polypeptides (DNA polymerase III) that were not part of the query (see Figure 5).

Once a query has been composed, the query engine can be launched and the sub-graph results can be further explored by using the Table and the Graph Riders. The Table Rider provides a tabular view of all the results. To this purpose, each sub-graph is linearized and associated to a line in the table. The columns correspond to the attributes of vertices and edges (Figure 6). The Table Rider therefore provides an overall view of the results and allows to select interesting lines (i.e. sub-graphs). Once a line is selected, the Graph Rider provides a graphic view of the corresponding sub-graph (Figure 7) and allows to browse the whole data graph by exploring the neighbours of displayed vertices.

#### Core API and tasks

Besides the graphical user interface, all operations of the GenoLink Core, such as graph creation, data import, graph querying and display, can be executed and controlled programmatically through a Java Core API. User-provided Java code can be dynamically loaded and executed in the system (this functionality is provided by an extension of the AROM system called AROM Tasks). This allows the user having some programming skills to design her/his own tasks to perform some specialized work. The core API and several tasks examples are distributed with the software's documentation.

#### Implementation

GenoLink, like all modules of the Genostar platform, is written in Java. It uses standard Java libraries such as Xerces (XML parser, [25]) and Xalan (extensible style sheet transformations (XSLT) engine [26]) from the Apache Software Foundation, Castor (Java to XML binding, [27]) from "Exolab.org" and GNU RegExp (regular expression pattern matching engine, [28]) from "Castor.org". The AROM task interpreter is built upon Beanshell [29]. All graphical user interfaces are written with the Java Swing library.

## Results and discussion

We now illustrate the querying capabilities of the system by investigating the functions of genes and proteins from bacteria *Escherichia coli *and *Helicobacter pylori*. To this purpose, we imported the following data: the complete genomes of the two bacteria (RefSeq entries NC000913 and NC000915) along with the annotations coming from COG [21], InterPro [23] and Enzyme Classification data [24]. The resulting data graph contains 21109 objects (proteins, genes, organisms, chromosomes, clusters of orthologous genes, etc.) linked together through 58064 relations. It takes about 5 minutes for GenoLink to read and to integrate the data into a single data graph occupying around 90 Mo in main memory (a data graph can be saved on disk for later reuse. In this example, it takes 30 seconds to reload the data graph from the saved binary file, which is 10 Mo in size).

Table 1 [see Additional file 1] displays several examples of queries addressing various biological questions in this context.

Query Q1 is a very simple example showing how to retrieve all CDS from *E. coli*. The constraint on the Organism reads as 'the Name attributes matches "coli"' and is indicated in italics below the Organism vertex.

Query Q2 illustrates how to search for *E. coli *'hypothetical' proteins that are annotated with the Enzyme Commission database. When this query is run against the complete data graph, 94 'hypothetical' proteins are found that are annotated with an EC number. Now, it is worth noting that the same query executed against a data graph containing only the *E. coli *RefSeq genome does not return any result. The difference illustrates the data integration process: the RefSeq *E. coli *genome has been dynamically augmented with new annotations (i.e. links between proteins and EC numbers) when we imported the EC database.

GenoLink can be used to refine a query, depending upon the results of a previous one. As an example, query Q3 retrieves all pairs of orthologous genes (according to the COG database) between *H. pylori *and *E. coli*. Query Q4 refines the previous one by restricting to genes coding for 'hypothetical' proteins.

Query Q5 illustrates how to retrieve all pairs of genes between *H. pylori *and *E. coli *encoding for proteins having a common InterPro domain.

Query Q6 shows how it is possible to handle negation. In this example we are interested in finding genes with no (COG) orthologs between the two species; by extension, we could suspect this genes are specific to each organism. To this purpose, GQL proposes a 'neighbours' operator. This operator explores the immediate neighbourhood of a vertex in a data graph and counts the number of vertices of a given type that are connected to it. In query Q6 that operator is used as follows: when the query engine examines vertex of type ProteinGene, it will count how many GeneOrtholog vertices are linked to that ProteinGene and will retain this vertex only if this number is 0 (therefore the ProteinGene has no orthologs).

Query Q7 gives an example of how some information can be inferred from one organism to the other. In this example we would like to infer unknown protein-protein interactions into *E. coli *from already known interactions in *H. pylori *and gene orthology relationships. To this purpose, we first load the complete protein-protein interactions map from *H. pylori *[30]. Then, we built up query Q7 under the assumption that if it exists an interaction between two proteins in *H. pylori*, and if these proteins are encoded by genes having (COG) orthologs in *E. coli*, then we may hypothesize that these *E. coli *proteins could interact as well. In this example, this yields 457 different answers, one of them is displayed in Figure 7. For this kind of query, the Table Rider (see section *User interfaces*) proved to be very useful since it provides a synthetic view allowing for a quick visual inspection of the 457 couples of possibly interacting proteins (Figure 6).

## Conclusion

GenoLink is a new software platform adding to existing DBMS new functionalities dedicated to the querying and exploration of data graphs. GenoLink handles graphs where vertices and edges are enriched with data modelled using an entity-relationship model. The platform provides the biologists with a rich visual environment to graphically explore genomic and post-genomic data, without prior knowledge of any programming or database querying languages. GenoLink provides the bioinformaticians with more advanced features such as a generic data graph exploration tool able to accommodate user-provided data models, a query language well-adapted to query graphs and a programming API.

## Availability and requirements

GenoLink is distributed either as a standalone application or as a component of the Genostar/Iogma platform. Both distributions are free for academic research and teaching purposes and can be requested at academy@genostar.com. A commercial licence form can be obtained for profit company at info@genostar.com. The distributions have been successfully tested on computers running RedHat Linux, Windows 2000/XP or MacOS X. The GenoLink distribution also incorporates a complete user's guide including a beginner's tutorial.

## Authors' contributions

PD and JW conceived the software architecture, designed the graph query language, the graph pattern matching algorithm and the whole set of graphical interfaces. PD managed the overall project, wrote most of the software code and documentation, and wrote the manuscript. LL and JLD participated in coding the graphical interfaces. AM and PD encoded the graph library used by GenoLink. AV and JW initiated the project in the Genostar consortium. All authors participated in the development of the data model used to investigate the functions of genes and proteins, in testing the software and in editing the manuscript.

## Supplementary Material

Additional File 1 GenoLink query graph examples. Each row corresponds to a different query (from simple to more sophisticated ones). The Query column gives an informal statement of the query, the Query-Graph column displays the corresponding graph pattern, that has to be constructed in the Query Builder (Figure 5). The Results column indicates the number of distinct results obtained (see text for information about the origin of data). The Time column indicates the execution time (in seconds). In the sake of clarity, the following code as been used to denote the type of edges: ILO for IsLocatedOn, IRO for IsRepliconOf, ICF for IsCodingFor, HPA for HasPolypeptideAnnotation, IIG for IsInGeneOrtholog, CD for ContainsDomain and HPIW for HasPhysicalInteractionWith. When applicable, constraints are displayed in italics under the concerned vertex or edge. Click here for file
