# Supplementary material for: GenoLink: a graph-based querying and browsing system for investigating the function of genes and proteins
Source: BMC Bioinformatics. 2006 Jan 17;7:21. doi: 10.1186/1471-2105-7-21 (PMC1382257; doi:10.1186/1471-2105-7-21)
Supplement: Additional File 1 — GenoLink query graph examples. Each row corresponds to a different query (from simple to more sophisticated ones). The Query column gives an informal statement of the query, the Query-Graph column displays the corresponding graph pattern, that has to be constructed in the Query Builder (Figure 5). The Results column indicates the number of distinct results obtained (see text for information about the origin of data). The Time column indicates the execution time (in seconds). In the sake of clarity, the following code as been used to denote the type of edges: ILO for IsLocatedOn, IRO for IsRepliconOf, ICF for IsCodingFor, HPA for HasPolypeptideAnnotation, IIG for IsInGeneOrtholog, CD for ContainsDomain and HPIW for HasPhysicalInteractionWith. When applicable, constraints are displayed in italics under the concerned vertex or edge. [file 1471-2105-7-21-S1.pdf]

	Query	Query-Graph	Results	Time (s)
Q1	Find all CDS from *E. coli*	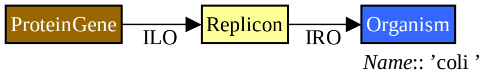	4279	< 1
Q2	Find all ‘hypothetical’ proteins from *E. coli* annotated with an EC number.	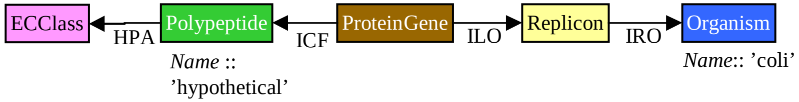	94	< 1
Q3	Find all orthologous genes between *E. coli* and *H.pylori*.	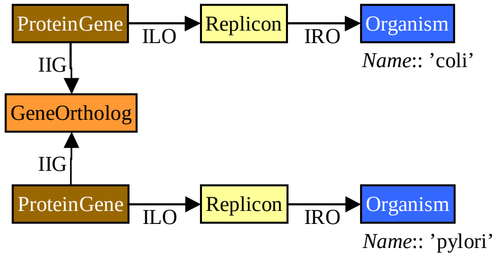	2301	29
Q4	Find all ‘hypothetical’ proteins from *H.pylori* that are orthologous to known proteins from *E. coli*.	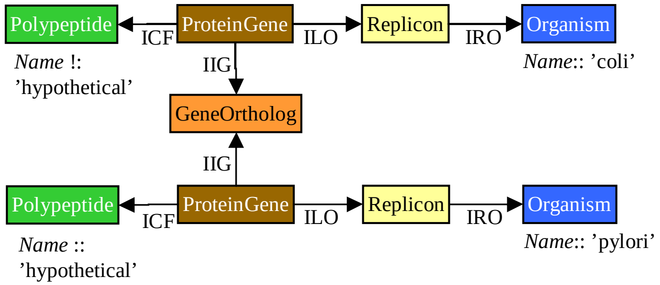	75	3
Q5	Find all proteins from *H.pylori* and *E.coli* having similar domain(s).	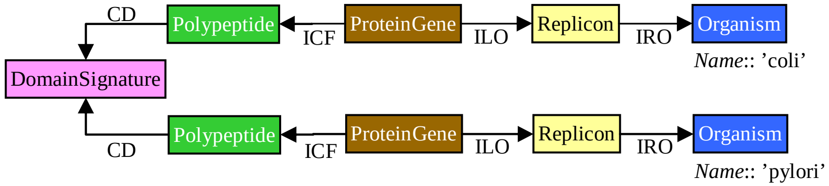	719	57

	Query	Query-Graph	Results	Time (s)
Q6	Find all genes coding for known protein that are specific to *H.pylori*.	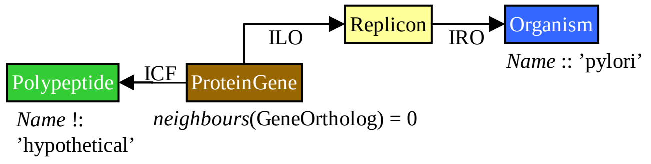	72	< 1
Q7	Infer protein-protein interactions from *H.pylori* into *E.coli*.	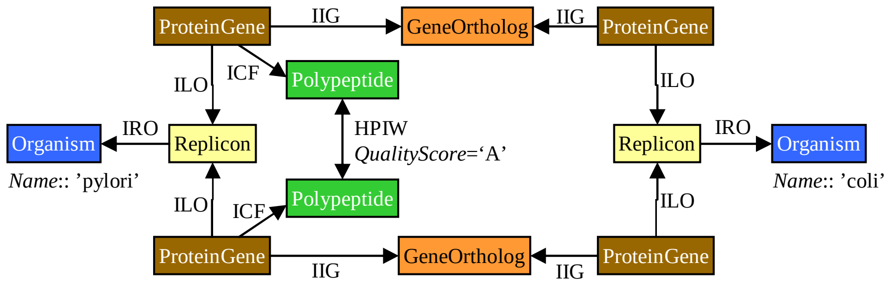	457	12
